# An Isolated Articular Surface Fracture of the Distal Patella: An Unusual and Previously Unreported Paediatric Injury

**DOI:** 10.1155/2013/605852

**Published:** 2013-12-08

**Authors:** Faiz S. Shivji, Darryl N. Ramoutar, James B. Hunter

**Affiliations:** Queen's Medical Centre, Derby Road, Nottingham NG7 2UH, UK

## Abstract

Paediatric patella fractures are uncommon, accounting for less than 1% of all paediatric fractures. This case report describes a previously undocumented patella fracture in a child, with a clear mechanism of injury. We present a case of a previously healthy 14-year-old boy who fell directly onto his right knee after coming off his pushbike. He sustained an isolated fracture involving the articular surface of the distal part of the patella with minimal displacement. The patient was managed conservatively in a Richard splint for three weeks, followed by a knee brace with gradually increasing degrees of flexion. He was instructed to be nonweight bearing for two weeks and then partial weight bearing for six weeks. At the final followup, after 9 weeks, the patient had full return of function and standard radiographs show the fracture to be healed. This case report has demonstrated how direct compression to the paediatric patella can cause a fracture isolated to its articular surface. It has detailed the natural progression of this injury to radiographic union, using a conservative management strategy. The authors believe that this case report provides an interesting insight into the variation of paediatric patella fractures and their contrasting management strategies.

## 1. Introduction

Paediatric patella fractures are uncommon, accounting for less than 1% of all paediatric fractures [1].

Previously documented patella fractures can be explained by mechanism of injury and the subsequent direction of force. Transverse fractures of the patella are usually caused by a direct force compressing the patella against the femur [2]. Valgus and lateral translational forces across the patellofemoral joint, such as when a child stands and leans to the contralateral side, are associated with osteochondral fractures with or without patella dislocation [3]. Sleeve fractures are avulsion type injuries whereby a small fragment of patella is pulled off with its cartilage, periosteum, and retinaculum [4].

This case report describes a previously undocumented type of patella fracture in a child, with a clear mechanism of injury. This report also details the management strategy utilised in the successful treatment of this type of fracture.

## 2. Case Presentation

**Figure 1:**
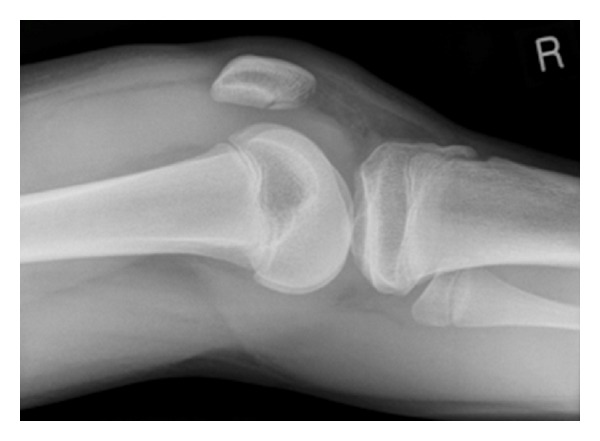
Lateral radiograph of the right knee taken on initial presentation.

A healthy 14-year-old boy came off his pushbike and fell directly onto his right knee. He immediately experienced pain in his knee that was worsened on weight bearing. This was also associated with significant swelling of his knee. He presented to the emergency department, where examination findings showed a significantly limited range of movement due to pain. A radiograph of his knee showed an isolated fracture of the articular side of the patella with a joint effusion (Figure 1). He was placed in a Richard splint, and a review in the next paediatric fracture clinic was arranged.

On review in fracture clinic three days after the injury, the patient had a significant effusion in his right knee. He was exquisitely tender over his patella and had an intact, though limited, straight leg raise. Range of movement was limited to 30 degrees of flexion, beyond which the pain was intolerable. An MRI was arranged to assess for any associated chondral damage suggesting osteochondritis dissecans, and to look for any other soft tissue derangement of the knee. The patient was treated for one week in a Richard splint and was given instructions to remain nonweight bearing.

**Figure 2:**
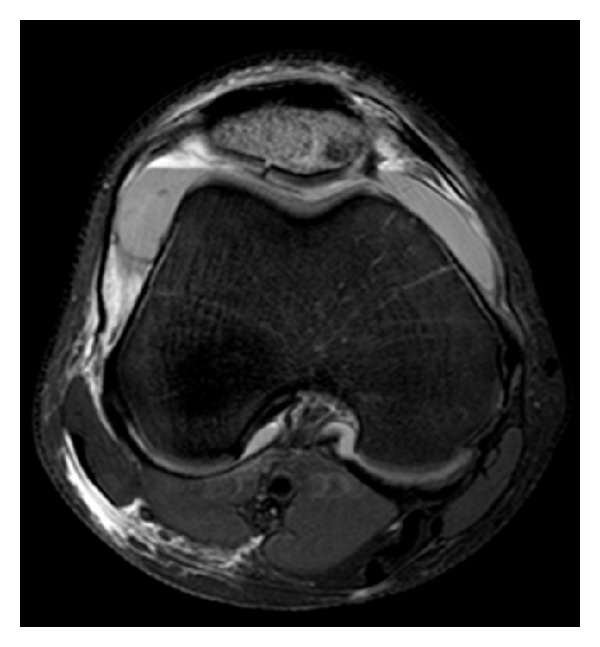
Axial T1 weighted magnetic resonance image taken one week after presentation.

**Figure 3:**
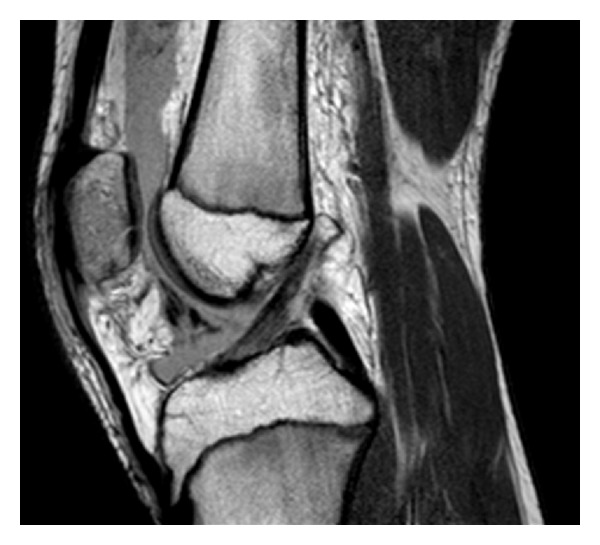
Sagittal T1 weighted magnetic resonance image taken one week after presentation.

Magnetic resonance imaging (MRI) of the knee (Figures 2 and 3) showed an isolated fracture involving the articular surface of the distal part of the patella with surrounding marrow oedema but minimal displacement. The overlying cartilage was intact except along the fracture line with no suggestion of osteochondritis dissecans patellar dislocation, or medial patellofemoral ligament rupture. The quadriceps mechanism was shown to be intact.

Ten days after the injury, the child still had a moderate effusion in his knee and was tender over his patella. Radiographs showed no displacement in the fracture. He was kept in his Richard splint for a further two weeks but allowed to be partial weight bearing.

At four weeks after injury, the patient was mobilising using his crutches. On examination, only a small residual effusion was noted and radiographs revealed though no further displacement, and the fracture line was still visible. He was able raise his leg straight but flexion was limited to 15 degrees. The patient was put in a knee brace at this visit, fixed to a range of 0–30 degrees with a plan to gradually increase the range of movement of the knee. He was also referred to physiotherapy.

**Figure 4:**
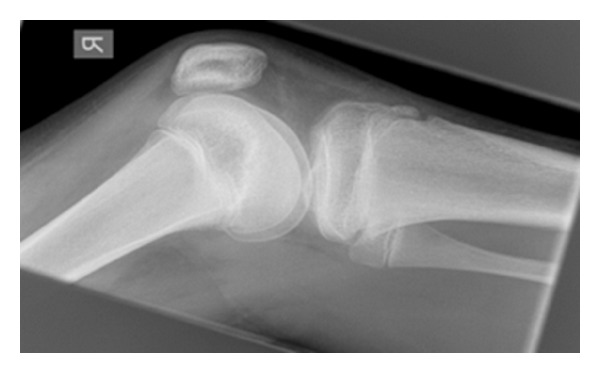
Lateral radiograph of the right knee taken seven weeks after injury.

At the fifth week review he had no tenderness over his patella and the effusion had resolved. He was able to flex his knee comfortably to 30 degrees in his brace. The degree of flexion in the brace was sequentially increased to 60 degrees, then 90 degrees and the patient was reviewed two weeks later (seven weeks after injury) at which point he could flex to 100 degrees without any discomfort. Radiographs taken showed that the fracture was healed (Figure 4). He was therefore allowed to fully mobilise without the brace.

At his final appointment, the 14-year-old boy was back to full function with an uncompromised range of movement in his knee and no residual pain.

## 3. Discussion

Known patella fracture patterns in children are those of transverse or comminuted fractures, osteochondral defects, and sleeve fractures [5–7]. Transverse fractures may be suffered after a direct compression force against the patella [6]. This case report shows how a direct compression force can also cause a fracture of solely the articular surface.

A clinician must have a high index of suspicion to diagnose a patella fracture, considering that the child may have experienced no direct trauma. On examination, children may have a knee effusion and painful knee flexion, features that were found in the patient in this case report [5]. An inability to raise his leg straight may also be present, especially in transverse fractures [6].

Plain film radiographs form the initial imaging process but may often miss osteochondral defects and sleeve fractures if the avulsed fragment consists mainly of cartilage [8]. Therefore, in a child with examination findings and a mechanism of injury as described, an MRI should be sought to confirm the diagnosis, as was done in this case report. Ultrasonography may provide an alternative diagnostic tool for sleeve fractures in the absence of MRI [9].

In this case report, MRI was used to exclude ligamentous damage and Osteochondritis Dissecans (OCD). OCD, described by König in 1887, is a disease affecting the articular cartilage and subchondral bone [10]. A disruption to the blood supply of the subchondral bone leads to avascular necrosis and subsequent bone resorption [7]. Articular cartilage may therefore become separated from the bone leading to osteochondral fragments in the joint space, resulting in pain and reduced movement. Therefore, OCD can be differentiated from simple fractures due to a different pathogenesis. Although both fractures and OCD may present in similar fashions, in OCD an underlying pathology exists.

An MRI was important to exclude OCD, as the management of this child may then have been different, for example, with the use of transchondral drilling [11].

The treatment of patella fractures is usually by open reduction and internal fixation. Various techniques have been used for transverse and sleeve fractures, such as tension band wiring and absorbable anchor sutures [5, 6, 12]. Osteochondral fractures can be treated by removal of the fragment via arthroscopy in addition to any treatment required for concomitant patella dislocation [13].

As the usual management for patella fractures is operative, especially those involving the articular surface, this case report shows a successful conservative approach for an unusual patella fracture. In this patient, splinting the knee and an inhibition of weight bearing for three weeks, followed by gentle increases in flexion and weight bearing, were shown to be sufficient for the fracture to heal. This method has also given an optimal functional outcome for this patient.

## 4. Conclusions

This paper has shown a rare type of patella fracture in a child caused by a direct compression mechanism of injury. It has detailed the natural progression of the articular surface fracture in this child to radiographic union, using a conservative management strategy. The authors believe that this case report provides an interesting insight into the variation of paediatric patella fractures and their contrasting management strategies.
